# Comparative Rice Bran Metabolomics across Diverse Cultivars and Functional Rice Gene–Bran Metabolite Relationships

**DOI:** 10.3390/metabo8040063

**Published:** 2018-10-09

**Authors:** Iman Zarei, Emily Luna, Jan E. Leach, Anna McClung, Samuel Vilchez, Ousmane Koita, Elizabeth P. Ryan

**Affiliations:** 1 Department of Environmental and Radiological Health Sciences, College of Veterinary Medicine and Biomedical Sciences, Colorado State University, Fort Collins, CO 80523, USA; iman.zarei@uef.fi; 2 Department of Bioagricultural Sciences and Pest Management, Colorado State University, Fort Collins, CO 80523, USA; emily.peachey@colostate.edu (E.L.); jan.leach@colostate.edu (J.E.L.); 3 USDA-Agricultural Research Service, Dale Bumpers National Rice Research Center, Stuttgart, AR 72160, USA; anna.mcclung@ars.usda.gov; 4 Center of Infectious Diseases, Department of Microbiology and Parasitology, Faculty of Medical Sciences, National Autonomous University of Nicaragua, León (UNAN-León), León 21000, Nicaragua; samuelvilchez@gmail.com; 5 Laboratoire de Biologie Moléculaire Appliquée, Campus de Badalabougou, Université des Sciences, des Techniques et des Technologies de Bamako, BP 1805 Bamako, Mali; okoita@icermali.org

**Keywords:** rice bran, rice genes, rice cultivars, metabolic pathway, metabolomics

## Abstract

Rice (*Oryza sativa* L.) processing yields ~60 million metric tons of bran annually. Rice genes producing bran metabolites of nutritional and human health importance were assessed across 17 diverse cultivars from seven countries using non-targeted metabolomics, and resulted in 378–430 metabolites. Gambiaka cultivar had the highest number and Njavara had the lowest number of metabolites. The 71 rice bran compounds of significant variation by cultivar included 21 amino acids, seven carbohydrates, two metabolites from cofactors and vitamins, 33 lipids, six nucleotides, and two secondary metabolites. Tryptophan, α-ketoglutarate, γ-tocopherol/β-tocopherol, and γ-tocotrienol are examples of bran metabolites with extensive cultivar variation and genetic information. Thirty-four rice bran components that varied between cultivars linked to 535 putative biosynthetic genes using to the OryzaCyc 4.0, Plant Metabolic Network database. Rice genes responsible for bran composition with animal and human health importance is available for rice breeding programs to utilize in crop improvement.

## 1. Introduction

Rice, a major global cereal crop, originates from *Oryza sativa* L. (Asian rice) or *Oryza glaberrima* Steud (African rice) [1]. *Oryza sativa* is the primary source of calories for half of the world’s population [2], and it provides phytochemicals, vitamins, minerals, carbohydrates, and fats when consumed as whole grain rice [3]. Rice grows in over 100 countries [4]. As a result of its long history of cultivation and selection under diverse environments, *O. sativa* comprises over 400,000 varieties and it has acquired a broad range of adaptability and tolerance to different water and soil regimens, from flooded lowlands to arid hillside slopes [5,6]. The 2017 global paddy rice production reported by the Food and Agriculture Organization (FAO) of the United Nations was 756.7 metric million tons, with over 502 million metric tons milled and more than 60 million metric tons of rice bran produced [7]. Polished white rice is the major product, with rice milling yields of 65–70% and the by-products comprising of 20% rice husk and 8–12% rice bran [8].

Rice bran has a broad spectrum of health benefits [9,10,11,12], partially due to a diverse array of bioactive metabolites [13,14]. Rice bran bioactive components include, but are not limited to γ-oryzanol [15], tocopherols, tocotrienols [16], carotenoids [17], γ-aminobutyric acid [18], octacosanol [19], squalene [20], unsaturated fatty acids [21], phytosterols, and phenolic compounds [22]. Dietary consumption of rice bran was shown to be feasible and tolerable to increase key nutrients and fiber intakes in children and adults [11,23,24,25], and to provide health-promoting properties in the prevention and control of major chronic diseases, such as diabetes [26], chronic inflammation [27], and cardiovascular disease [25,28], as well as cancers of the colon, liver, prostate, and breast [11,24,29,30]. Until recently, rice bran has been largely under-valued and under-utilized for nutritional and medicinal applications [31], and this promising food has not received attention from rice breeders when compared to other traits of agronomic importance (e.g., yield, disease resistance) [32]. Given that the bran component of whole grain rice is the fraction with highest nutritive value [33,34], and that extensive genetic variation exists in *Oryza sativa* germplasm, a continued exploration with regards to the nutritional and health properties of rice bran is merited. 

Metabolomics has shown the utility to study rice plant biology and compounds that are linked to tolerance to different stressors, including abiotic stress [35], mineral toxicity [36], nutrient limitation [37,38], drought stress [39], and pesticide stress [40], suggesting extensive metabolome adaptability in rice. Metabolomics has also characterized natural and genetic variations in rice via the phenotyping of brown rice seeds [41], cooked brown rice [42], mature seeds [43], embryo (a fraction of rice bran) and endosperm (white rice) [44], and leaves [39]. These findings support metabolomic approaches to improve plant function, enhance grain nutritional quality, and increase grain yield [6,45,46]. Metabolite profiling of rice bran from three USA rice cultivars showed an appreciable variation of bioactive rice bran components, and provided the rationale for larger, global scale investigations [13,47]. Variation in bran composition could substantially contribute to greater interest in whole grain rice for wider consumer acceptance.

There is a gap in knowledge between rice bran bioactive metabolites and their genetic variation among globally diverse rice cultivars. Given that metabolomics is a powerful tool that allows for a broad range of rice bran metabolite detection, the objective of this study was to investigate variation in the rice bran metabolome of 17 cultivars, and to identify gene–metabolite relationships that are relevant to the nutritional and medicinal qualities of rice bran. Rice bran metabolites were hypothesized to exhibit biochemical variation across cultivars and reveal key rice gene–metabolite relationships of utility for rice bran traits with nutrition and health importance in future breeding programs.

## 2. Results

### 2.1. Classification of Rice Bran Metabolite Profiles and Metabolic Pathways by Non-Targeted Metabolomics

The rice bran metabolome ranged in the total numbers of identified metabolites, with 378 metabolites identified in Njavara (India) rice bran, to 430 metabolites identified in Gambiaka (Mali) rice bran. Table 1 shows the rice bran metabolite numbers based on chemical classes and shows the cultivar(s) that had the highest and lowest total number of metabolites. RBT 300 had 122 amino acids, and Khao Gaew and Njavara cultivars had 99 amino acids. Gambiaka, Shwetasoke, and LTH had 53 carbohydrates, while Calrose had the smallest total number, with 48 carbohydrates. On average, 39% of the rice bran metabolome were lipids across the 17 cultivars, and this represented the largest composition by chemical class. Basmati 217 and Shwetasoke had the largest numbers of lipids (166 total), whereas Jasmine 85 had the lowest number of lipids (146 total). For the nucleotides, Basmati 370 and Sawa Mahsuli had 38 metabolites, and Khao Gaew and Njavara had 29. Overall, the “red color” bran genotypes, i.e. Njavara and LTH, had a broader range of secondary metabolites (22 metabolites), while Dorado, having brown bran, had 16 metabolites. Cultivars ranged from zero to 11 metabolites in rice bran peptides. It is noteworthy that the number of cofactors and vitamins did not vary greatly across rice bran cultivars (23–27 metabolites) (Table 1 and Supplementary Table S1). Each of the chemical classes were subdivided into 53 metabolic pathways (Supplementary Table S1).

### 2.2. Global Rice Bran Metabolome Variation for 17 Cultivars

**Figure 1:**
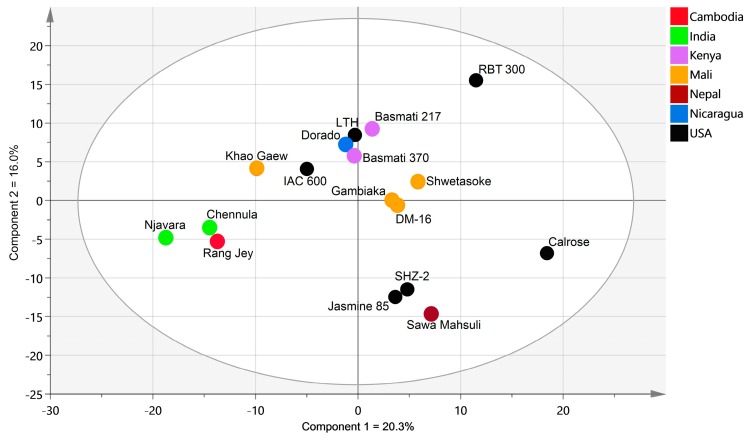
Principal component analysis (PCA) of rice bran metabolome for 17 rice cultivars. PCA was completed using median-scaled relative abundance of all bran in the 17 rice cultivars. The PC1 showed 20.3% variation, and PC2 showed 16% variation in the metabolite profiles. Colored dots indicate the country where the rice was produced.

Across all cultivars, the principal component 1 (PC1) accounted for 20.3% of the total variation in the dataset, and PC2 for 16% of the metabolite variation (Figure 1). These results indicate that there is a “core” rice bran metabolome, and that the difference in the range of individual rice bran metabolites (ca. 60–90) can be seen across the different chemical classes of the metabolome.

### 2.3. Comparison of Rice Bran Metabolites across 17 Cultivars

**Figure 2:**
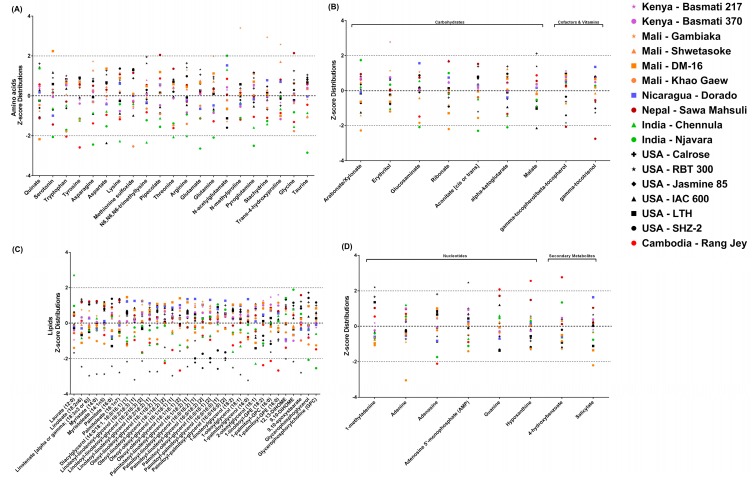
Comparison of discriminating 71 rice bran metabolites across 17 cultivars and based on the Z-scores obtained from the relative abundance of each metabolite. The 21 amino acids (**Panel A**), seven carbohydrates and two cofactors and vitamins (**Panel B**), 33 lipids (**Panel C**), six nucleotides and two secondary metabolites (**Panel D**) have Z-scores expressed as standard deviations from the mean and were calculated using the following formula: Z = (*x* − µ)/σ, “*x*” is relative abundance of the metabolite, “µ” is mean of relative abundance for the metabolite across 17 rice brans, and “σ” is the relative abundance standard deviation of same metabolite across 17 cultivars. Metabolites above 2 or below −2 in the panel A–D are highlighted for the largest variation, and noted by cultivar and the country where the rice was grown. Colored symbols are associated with the location where the rice was grown.

Z-score was used to represent the median-scaled relative abundance of each rice bran metabolite across all cultivars, and a threshold Z-score of |2| was applied to identify metabolites that differed appreciably between cultivars. This comparative Z-score analysis led to the identification of 71 rice bran metabolites that were significantly different across cultivars. The cultivar discriminating metabolites are shown in Figure 2 (panel A–D), and complete metabolite characterizations are included in Supplementary Table S2. Thus, the 20% variation identified using comparative rice bran metabolomics (PC1) included 21 amino acids, seven carbohydrates, and two cofactors & vitamins, 33 lipids, six nucleotides, and two secondary metabolites. 

#### 2.3.1. Cultivar Variation in Rice Bran Amino Acids

There were a total of 21 amino acids that differed among cultivars, and among these, many metabolites had previously-reported roles in human and animal health. Figure 2A shows that DM-16 rice bran had lower and higher expressions of quinate and serotonin, respectively. Rang Jey showed the lower Z-score for tryptophan and tyrosine. Chennula rice bran had lower relative abundances of four amino acids, namely lysine, *N6*,*N6*,*N6*-trimethyllysine, threonine, and arginine. Gambiaka rice bran showed higher relative abundances of *N*-methylproline, stachydrine, and trans-4-hydroxyproline when compared to other brans, while Njavara was lower in the abundances of serotonin, asparagine, glutamate, glutamine, pyroglutamine, and taurine. Njavara was higher in relative abundances of *N*-acetylglutamate. Higher abundances of pipecolate and glycine were observed in Sawa Mahsuli among the cultivars. Khao Gaew and IAC 600 were the only cultivars with a lower relative abundance of methionine sulfoxide and aspartate, respectively. Basmati 217 and Basmati 370, Shwetasoke, Dorado, Calrose, RBT 300, Jasmine 85, LTH, and SHZ-2 were the cultivars with no significant Z-score changes across the class of amino acids. 

#### 2.3.2. Cultivar Variation in Rice Bran Carbohydrates

Seven rice bran carbohydrates showed significant differences across cultivars (Figure 2B). Cultivars with lower abundances is some metabolites included arabonate/xylonate in Khao Gaew, glucosaminate, aconitate, and α-ketoglutarate in Njavara, and malate in IAC 600. Calrose was higher for the abundance of malate, while Basmati 217 had the highest abundance of erythritol. The remaining cultivars (i.e., Basmati 370, Gambiaka, Shwetasoke, and DM-16, Dorado, Sawa Mahsuli, Chennula, RBT 300, Jasmine 85, LTH, SHZ-2, and Rang Jey) showed no significant Z-score changes across the carbohydrate chemical class. 

#### 2.3.3. Cultivar Variation in Rice Bran Cofactors and Vitamins

There was limited variation for cofactors and vitamins in bran amongst the cultivars, except for the vitamin E components γ-tocopherol/β-tocopherol and γ-tocotrienol, with the lowest relative abundances observed in Sawa Mahsuli rice bran (Figure 2B). 

#### 2.3.4. Cultivar Variation in Rice Bran Lipids

Lipids represented the largest percentage of the bran metabolome (39%) encompassing 146-166 metabolites across varieties (Table 1). Figure 2C shows the 32 lipid metabolites with significant Z-score below -2 and one lipid was significantly higher in abundance (Z-score above 2) when compared across cultivars. Chennula was the only cultivar that had an increased Z-score for laurate. RBT 300 (20 lipids), Rang Jey (five lipids), Jasmine 85 (four lipids), Njavara (two lipids), and Chennula (two lipids) were the cultivars with lower metabolite relative abundances. The significant metabolites in RBT 300 included linoleate, linolenate, myristate, myristoleate, palmitate, palmitoleate, diacylglycerol (14:0/18:1, 16:0/16:1), two isomers of linoleoyl-linolenoyl-glycerol (18:2/18:3), linoleoyl-linoleoyl-glycerol (18:2/18:2), oleoyl-linoleoyl-glycerol (18:1/18:2), oleoyl-oleoyl-glycerol (18:1/18:1), palmitoleoyl-linoleoyl-glycerol (16:1/18:2), palmitoyl-palmitoyl-glycerol (16:0/16:0), 1-linoleoylglycerol (18:2), 1-oleoylglycerol (18:1), 1-palmitoylglycerol (16:0), 12,13-dihydroxyoctadec-9-enoic acid (12,13-DiHOME), 9,10-DiHOME, and 9,10-epoxystearate. Rang Jey was lower in bran oleoyl-linoleoyl-glycerol (18:1/18:2), oleoyl-oleoyl-glycerol (18:1/18:1), 1-linoleoyl- glycerophosphoethanolamine (GPE) (18:2), 1-palmitoyl- glycerophosphocholine (GPC) (16:0), and 1-palmitoyl-GPE (16:0). Jasmine 85 showed a lower relative abundance of two isomers of palmitoyl-linoleoyl-glycerol (16:0/18:2), palmitoyl-oleoyl-glycerol (16:0/18:1), and palmitoyl-palmitoyl-glycerol (16:0/16:0). Lower relative abundance lipids in Njavara included glycerophosphoglycerol and GPC. Chennula showed low abundances in oleoyl-linoleoyl-glycerol (18:1/18:2) and 2-oleoylglycerol (18:1). 

#### 2.3.5. Cultivar Variation in Rice Bran Nucleotides

Six nucleotides varied among the cultivars. Figure 2D shows that Rang Jey rice bran had higher abundance of guanine and hypoxanthine, and the lower abundance of adenosine when compared to the other rice cultivars. RBT 300 had higher relative abundance in 1-methyladenine and adenosine 5′-monophosphate (AMP). Khao Gaew showed a lower level of adenine amongst the cultivars. 

#### 2.3.6. Cultivar Variation in Rice Bran Secondary Metabolites

Secondary metabolites that were expressed differently among the cultivars included two metabolites: 4-hydroxybenzoate and salicylate. Figure 2D shows that Rang Jey had the highest abundance of 4-hydroxybenzoate, and Khao Gaew had the lowest relative abundance for salicylate when compared to all other cultivars. There were no differences in the relative abundances of rice bran peptides, yet there was cultivar variation in the presence or absence of the rice bran peptides (0–11 metabolites), whereby Basmati 217 was the only rice bran that had no peptides identified. Chennula, Njavara, and RBT 300 had one identified peptide (prolylglycine in Chennula and Njavara, and valylglutamine in RBT 300). There were seven cultivars with 11 peptides identified (Gambiaka, Shwetasoke, DM-16, Sawa Mahsuli, Calrose, Jasmine 85, and SHZ-2). 

### 2.4. Integrating Rice Biosynthetic Genes with Rice Bran Metabolites

Using OryzaCyc database, 34 out of the 71 cultivar-discriminating metabolites had corresponding gene(s) identified in the database. Rice genes listed in Table 2 were identified for 16 amino acids that differed across cultivars, and that were in the PMN databases for biosynthesis. Quinate and serotonin had five genes and two genes, respectively, which could regulate levels in the bran. Glycine is another amino acid with eight genes that were directly involved in its biosynthesis, yet there were 151 other possible rice genes in the database that were indirectly involved in glycine biosynthetic pathways. Lipids were the majority of the methanol-extracted rice bran metabolome, and of the 33 differentially-abundant rice bran lipids, only eight had biosynthetic genes characterized (Table 2). Glycero-phosphocholine (GPC) was found to have seven biosynthetic genes, whereby linolenate, an omega-3 polyunsaturated fatty acid (PUFA) was linked to only one gene, but it was indirectly associated with 332 genes that are common in biosynthesis of a standard fatty acid. Table 3 lists three carbohydrates and two cofactors and vitamins metabolites with registered biosynthetic genes. There were two genes responsible for malate biosynthesis, with an additional 150 genes that are common to the biosynthesis of non-specified carbohydrates. Tocochromanols, also known as Vitamin E components (tocopherols and tocotrienols, collectively), have genes in rice that are clearly defined [48], and γ-tocopherol/β-tocopherol had four genes, while γ-tocotrienol had a single known gene. There were four nucleotides and one secondary metabolite with rice genes reported for biosynthesis (Table 4). Salicylate had three identified biosynthetic genes, and another 152 possible genes may be involved in carboxylate biosynthesis.

### 2.5. Integration of Rice Bran Metabolites, Metabolic Pathways, and Rice Genes

**Figure 3:**
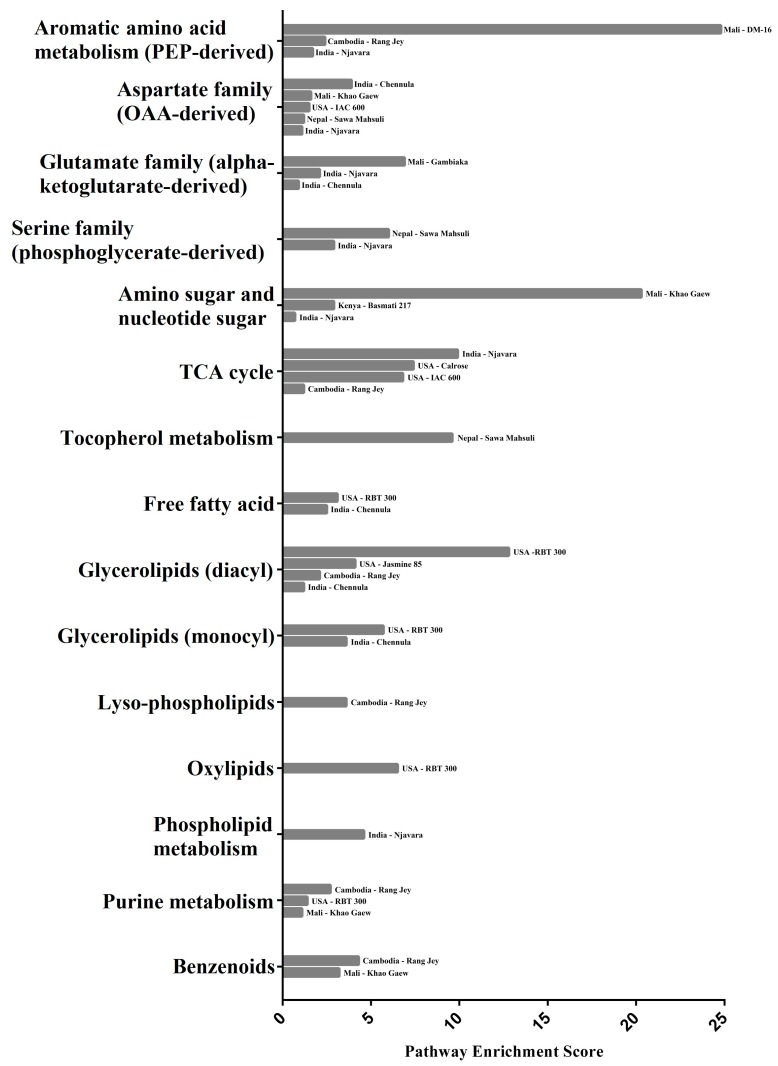
A pathway enrichment score (PES) of 15 metabolic pathways vary among the cultivars tested. Metabolic pathways with enrichment scores of greater or less than 1 are shown. These are for cultivars that contained one or more metabolites with a statistically significant Z-score.

Figure 3 shows the Pathways Enrichment Score (PES) for 15 metabolic pathways in cultivars that contained one or more metabolites with significant Z-scores among the 71 discriminating rice bran metabolites. A complete list of all PES is shown in Supplementary Table S3. 

#### 2.5.1. Amino Acid Metabolic Pathway Enrichment Scores and Gene Associations

Amino acids contained four metabolic pathways with PES distinctions, including aromatic amino acids (PEP-derived), aspartate family (OAA-derived), glutamate family (α-ketoglutarate-derived), and serine family (phosphoglycerate-derived), which involve the 21 amino acids that differed among the cultivars, including four aromatic amino acids (PEP-derived), seven aspartate family (OAA-derived), nine glutamate family (α-ketoglutarate-derived), and two serine family (phosphoglycerate-derived), as shown in Supplementary Table S2. DM-16 rice bran had low and high abundances of quinate and serotonin, respectively, which had the highest PES for the aromatic amino acid (PEP-derived) pathway (PES = 24.8). Chennula rice bran had the highest PES for the aspartate family (OAA-derived) pathway (PES = 3.9), and the low relative abundances of three amino acids, namely lysine, N6,N6,N6-trimethyllysine, and threonine explained this difference. Gambiaka rice bran had the highest PES for the glutamate family (α-ketoglutarate-derived) pathway (PES = 6.9) and a high relative abundance of N-methylproline, stachydrine, and trans-4-hydroxyproline explained this difference. Sawa Mahsuli rice bran had the highest PES for the serine family (phosphoglycerate-derived) pathway (PES = 6.0) when compared to the other cultivars. Differentially higher abundances of glycine in this cultivar were the contributors to this difference (Figure 3). 

#### 2.5.2. Carbohydrate Metabolic Pathway Enrichment Scores and Gene Associations

The carbohydrate chemical class contained two metabolic pathways with significant PES, and included amino sugars and nucleotide sugars, and the TCA cycle. Seven discriminating metabolites explained these changes, including four amino sugars and nucleotide sugars, and three TCA cycle metabolites. Rice genes involved in the biosynthesis of metabolites under the TCA cycle metabolic pathways are shown in Table 3. There were no genes identified for metabolites under the amino sugar and nucleotide sugar metabolic pathways. Khao Gaew rice bran had the highest PES for the amino sugar and nucleotide sugar pathway (PES = 20.3), and this was due to the low relative abundance of arabonate/xylonate and ribonate. Njavara rice bran had the highest PES for the TCA cycle across all cultivars (PES = 9.9) due to glucosamine, aconitate, and α-ketoglutarate having the lowest abundances among the cultivars (Figure 3). 

#### 2.5.3. Cofactors and Vitamins Metabolic Pathway Enrichment Scores and Gene Associations

Cofactors & vitamins were associated with the tocopherol metabolic pathway, whereby the Sawa Mahsuli rice bran had the highest score (PES = 9.6) due to a lower relative abundance of γ-tocopherol/β-tocopherol and γ-tocotrienol, as compared to other cultivars (Figure 3). Rice genes involved in the biosynthesis of metabolites under the tocopherol metabolic pathway are shown in Table 3.

#### 2.5.4. Lipids Metabolic Pathway Enrichment Scores and Gene Associations

The 33 lipid metabolites that explained these changes in PESs included seven free fatty acids, 14 glycerolipids (diacyl), four glycerolipids (monoacyl), three lyso-phospholipids, three oxylipins, and two phospholipids. Rice genes involved in the biosynthesis of metabolites under free fatty acids, oxylipins, and phospholipid pathways are shown in Table 2. There were no genes that were identified for metabolites under the monoacyl and diacyl glycerolipids, and lyso-phospholipid metabolic pathway. RBT 300 had the lowest relative abundance for the majority of lipids (20 lipids), which explain the highest PES for the free fatty acid pathway (PES = 3.1), glycerolipids (diacyl) pathway (PES = 12.8), glycerolipids (monoacyl) pathway (PES = 5.7), and oxylipins pathway (PES = 6.5). Rang Jey rice bran had the highest PES for the lyso-phospholipids pathway (PES = 3.6), and low abundances of 1-linoleoyl-glycerophosphoethanolamine (GPE) (18:2), 1-palmitoyl-GPC (16:0), and 1-palmitoyl-GPE (16:0) explain this significance. Njavara rice bran was another cultivar with highest PES for phospholipid metabolism (PES = 4.6), and a low abundance of glycerophosphoglycerol and GPC explain this significance (Figure 3). 

#### 2.5.5. Nucleotide Metabolic Pathway Enrichment Scores and Gene Associations

Within the nucleotides, a PES of 2.7 was identified for purine metabolism, with Rang Jey rice bran having the highest score. This PES was the result of a lower relative abundance of adenosine in this cultivar, compared to others (Figure 3). Rice genes involved in the biosynthesis of metabolites under the purine metabolic pathway are shown in Table 4.

#### 2.5.6. Secondary Metabolite Pathway Enrichment Scores and Gene Associations

Secondary metabolites that were expressed differently among the cultivars included two benzenoid pathway metabolites, namely, 4-hydroxybenzoate and salicylate. Rang Jey had the highest relative abundance for 4-hydroxybenzoate, and Khao Gaew had the lowest relative abundance for salicylate; thus the highest pathway enrichment score for Rang Jey (PES = 4.3), followed by Khao Gaew (PES = 3.2) (Figure 3). Rice genes involved in the biosynthesis of salicylate are shown in Table 4. No genes were identified for 4-hydroxybenzoate in the database.

## 3. Discussion

This comparative global rice bran metabolomics investigation led to the high throughput and sensitive identification of over 450 diverse rice bran metabolites/phytochemicals. Rice bran metabolites stem from multiple chemical classes, and metabolic pathways involved in rice seed development. The rice bran metabolomics analysis of 17 global cultivars revealed substantial variation in both the presence and abundance of metabolites from distinct chemical classes (~36% of entire metabolite profile), as well as from a ‘core’ rice bran metabolite profile (~74% of metabolites). Rice bran has been shown to be feasible for dietary use [11,23,49], and it is a valuable nutritional addition to polished rice, especially in the geographic regions where rice is one of the primary food sources. Moreover, rice is a staple crop in many low–middle-income countries where malnutrition remains a major problem [50,51]. Evaluating the nutritional and health benefits of rice bran is enhanced by using a global metabolomics approach. The 71 discriminating rice bran metabolites that varied among these 17 cultivars that were collected from diverse field environments should be considered in breeding, due to the presence of nutritionally- and medicinally-valuable bran metabolites. 

A few examples of amino acids with health benefits that were identified herein are quinate and serotonin from the aromatic amino acid metabolic pathway (PEP-derived) that has been shown to be anti-inflammatory [52] and antiemetic [53], respectively. This metabolome analysis showed that the abundance of quinate and serotonin were significantly lower and higher, respectively, in DM-16 (produced in Mali, West Africa) when compared to other rice brans. An increased relative abundance of glycine and pipecolate was also observed in Sawa Mahsuli (produced in Nepal). Glycine was shown to have anti-diarrheal [54], anti-inflammatory [55], antioxidant [56], cancer chemoprevention [57], and anti-obesity properties [58]. Pipecolate was shown to have cancer chemoprevention properties, as this phytochemical serves as a precursor to gut microbial secondary metabolites production, such as such as rapamycin, swainsonine, virginiamycin, and marcfortine that exhibit anti-inflammatory, antitumor, and antibiotic properties [59]. 

Carbohydrates, vitamins, and lipids of interest to improve animal and human health, differed in abundance across cultivars. Malate, a carbohydrate from the TCA cycle, was detected, and it had a higher relative abundance in Calrose (USA grown) when compared to other cultivars. In the human body, malate is important to energy metabolism during both aerobic and anaerobic conditions [60,61], and it was shown that a deficiency of malate may be a major cause of physical exhaustion [61]. Malate has also been reported to have natural antimicrobial activity against *Salmonella typhimurium* and other microbial pathogens [62]. Given that rice bran, and in particular the Calrose cultivar, is a good source of malate, it could be used to replenish the endogenous malate in the body, and warrants further attention for nutritional and breeding programs.

The Sawa Mahsuli cultivar from Nepal showed lower relative abundance of γ-tocopherol and γ-tocotrienol when compared to other cultivars, and it should be considered as a genetic resource for crop improvement, because total vitamin E contents found in rice bran are associated with health benefits [63]. γ-tocopherol, the primary form of vitamin E in food in the USA [64], and γ-tocotrienol, a safe and well-tolerated form of vitamin E [65], have demonstrated a broad range of disease fighting activities, including but not limited to anti-inflammatory [66] and anti-hypertension actions [67].

In the context of lipid metabolites, two bran sources that demonstrate the value of nutritional breeding considerations are RBT 300 (USA derived) and Jasmine 85 cultivar (USA grown). RBT 300, a blend of rice bran from California where Calrose cultivars predominate, was shown to be lower in the relative abundance of lipids, including linolenate (alpha or gamma), an essential fatty acid (omega-3 or omega-6, respectively), which is not synthesized by mammals [68]. Jasmine 85 was also shown to be low in relative abundance of four diacylglycerolipids, namely, two isomers of palmitoyl-linoleoyl-glycerol (16:0/18:2), palmitoyl-oleoyl-glycerol (16:0/18:1), and palmitoyl-palmitoyl-glycerol (16:0/16:0). These diacylglycerols are important to the diet because they deliver two distinctly important fatty acids. For example, palmitoyl-linoleoyl-glycerol (16:0/18:2) consists of palmitic acid and linoleic acid. Palmitic acid exerts multiple fundamental biological functions [69]. On the other hand, linoleic acid has been shown to be anticarcinogenic [70], and it reduces the risk of atherosclerosis in rabbits [71]. α-linoleic acid (ALA) acts as the precursor of eicosapentaenoic acid (EPA) and docosahexaenoic acid (DHA) [72]. Thus, ALA from rice bran may have as many beneficial effects as EPA and DHA to promote human health. Studies on humans and rodents have shown that the synthesis of anti-inflammatory prostaglandin E1 was selectively elevated through γ-linoleic acid supplementation [73]. Future studies should consider using rice bran lipids from bran or whole grain rice, as they may demonstrate beneficial effects when compared to consuming these lipids as isolated supplements. 

Other metabolites from this study that varied by cultivar, and that should be considered for importance to human health, were 4-hydroxybenzoate and salicylate. The 4-hydroxybenzoate had a higher relative abundance in Rang Jay (grown in Cambodia), and this compound has established antimicrobial properties [62,74], as well as antioxidant [75] actions. Salicylate, a phytochemical with a broad function in plant growth and development [76], is also an active component of aspirin, and it has well-documented anti-diabetic, anti-inflammatory, and cardio-protective properties [77,78]. It has the potential to be improved through selective breeding programs in Khao Gaew (from Mali), as salicylate had the lowest degree of abundance in this cultivar.

The rice bran phytochemical diversity that was observed among 17 cultivars produced in geographically diverse field environments included bran molecules that were present at very low levels, and which have been typically overlooked in targeted studies. For example, taurine was only recently shown to exist in rice bran [13], and this study verifies its lower abundance across cultivars and regions. Taurine is a known antioxidant and an anti-inflammatory agent, as well as a powerful scavenger of hypochlorous acid [79,80]. These effects of taurine may be also related to the prevention of obesity by increasing the energy metabolism in white adipose tissue [81]. In other studies, taurine was found to have antiepileptic actions [82], and to be neuroprotective against glutamate excitotoxicity [83]. 

Understanding the genes involved in biosynthesis of bran specific metabolites that are distinct from the rest of the grain is essential to improve nutritional and medicinal value of the bran and the whole grain (brown) rice. Increasing our understanding of the rice genes involved in the biosynthetic pathways of bran composition, such as lipid biosynthetic pathways are noteworthy, as these genes were largely missing from the integrated database (Table 3). Enhancing the rice bran amino acid and lipid contents, as well as many other phytochemicals, such as recently shown for tricin [84], may be beneficial to co-develop with high-yielding rice cultivars. Genome-wide association study (GWAS) using cultivar diversity panels [85,86] and quantitative trait locus (QTL) mapping in structured bi-parental populations [6,87,88], when coupled with rice bran metabolomics, offer a novel means of gene discovery and crop improvement. This emerging field of phytochemical genomics, integrating genomic, proteomic, and metabolomic approaches, has been used in crop improvement for barley [89], corn [90,91], potato [92], and tomato [93,94]. We put forth that the study and identification of rice genes underlying the nutritional and medicinal traits in rice bran deserve investigation to realize the tremendous potential impact of rice bran in global nutritional security.

Rice bran variation in the composition of bioactive molecules has functional relevance to rice breeders that seek to improve bran for human and animal health. The results of this study support the feasibility that numerous phytochemicals that have reported medicinal mechanisms of action can be improved in rice bran through breeding [13,14,95]. Rice bran, regardless of varietal differences, has a valuable ‘core’ metabolome, as well as a variable set of metabolites that differ among cultivars, which can be developed as an affordable food ingredient for a diverse global population that remains challenged to meet basic nutritional security needs. The identification of 71 metabolites and the ~1500 rice genes associated with the 15 metabolic pathways are significant results obtained herein that distinguish *Oryza sativa* cultivars and that support the use of bran small molecules as bio-markers. The current understanding of the genetic basis for the type and quantity of metabolites, and the metabolic pathways that exist in rice bran is sufficient to start breeding rice cultivars that contain optimal profiles for some rice bran metabolites that will benefit animal and human health. 

## 4. Materials and Methods

### 4.1. Rice Cultivars and the Heat Stabilization of Bran

Rice bran was isolated from 17 rice cultivars that originated from 11 countries and that were grown in seven countries including Cambodia, India, Kenya, Mali, Nepal, Nicaragua, and the United States, all having emerging interests in producing functional foods [9,10,23]. The phenotypes of all cultivars are described in Table 5. All the samples were stored as rough rice (paddy rice) until milling, to prevent any nutrient and chemical changes. Furthermore, the rice grain was milled using a Yamamoto test whitening machine Rice pal VP-31 T grinder and milling system. The milling process was performed at room temperature with 12% bran removal from the brown rice. Immediately after the milling, raw rice bran was heat-stabilized (110 °C for 6 min) to prevent rancidity, and then stored at −20 °C until further processing for metabolomics.

### 4.2. Rice Bran Metabolite Extraction and Sample Preparation

Metabolon Inc. (Durham, NC, USA) performed the global, non-targeted metabolomics. Prior to extraction, 10 recovery standards (i.e., quality controls) were added into the samples. Rice bran samples were extracted as previously described [13]. Briefly, following cryo-ground processing, all rice bran samples containing 100 mg (±2 mg) as a powder were cryo-weighted into 1.5 mL microcentrifuge tubes, and 80% methanol was added. Samples then underwent vigorous shaking for 2 min (Glen Mills GenoGrinder 2000), followed by centrifugation to precipitate the protein and to separate the small molecules from the macromolecules. The attained supernatant extract (i.e., rice bran extract) was divided into four fractions for different modes of analysis by ultrahigh performance liquid chromatography-tandem mass spectroscopy (UPLC-MS/MS), including reverse-phase chromatography with positive/negative ion mode electrospray ionization for non-polar compounds, and hydrophilic-interaction chromatography (HILIC) UPLC-MS/MS with positive/negative ion mode electrospray ionization for the analysis of polar compounds. Prior to injection, samples were placed on TurboVap^®^ (Zymark, Hopkinton, MA, USA) evaporator to make sure that there is no organic solvent remaining. 

### 4.3. UPLC-MS/MS Analysis

The non-targeted metabolomics analysis was based on previously described methods [13]. Briefly, a Waters ACQUITY UPLC coupled with a Thermo Scientific Q-Exactive high resolution/accurate mass spectrometer interfaced with a heated electrospray ionization (HESI-II) source and Orbitrap mass analyzer was utilized. The dried rice bran extract was reconstituted in UPLC-compatible solvents (acidic or basic solvents) for each mode of analysis. For the acidic solution, the rice bran extract was analyzed once for hydrophilic compounds, and once for hydrophobic compounds. For hydrophilic compounds, the extract was eluted from a C18 column (Waters UPLC BEH C18-2.1 × 100 mm, 1.7 µm) using water and methanol, containing 0.05% perfluoropentanoic acid and 0.1% formic acid in a gradient manner. For the hydrophobic compounds, the extract was gradient-eluted from the same C18 column and solvent mentioned, with added acetonitrile.

Similar to the acidic mode, in the basic solution, rice bran extract was once analyzed for hydrophilic compounds and once for hydrophobic compounds using a similar C18. For more hydrophobic and positive ion compound extraction, water, methanol, and 6.5 mM ammonium bicarbonate at pH 8.0 was used to elute the rice bran extracts from the C18 column. For more hydrophilic and more negative ion compounds, the extract was analyzed and eluted through an interaction liquid chromatography (HILIC) column (Waters UPLC BEH Amide 2.1 × 150 mm, 1.7 µm) using a gradient consisting of water and acetonitrile and 10 mM ammonium formate, at pH 10.8. Using dynamic exclusion, the mass spectrometry analysis was interchanged between MS and data-dependent MS^2^ scans with a scan range of 70–1000 *m/z*.

### 4.4. Metabolite Data Extraction and Compound Identification

Biochemical identifications from UPLC-MS was completed using the Metabolon in-house developed peak detection and integration software based on three criteria: retention index, accurate mass match to the National Institute of Standards and Technology library within ±0.005 atomic mass units, and tandem mass spectrometry (MS/MS) scores between the generated data from the experiment and standards. This software uses standard industry approaches for MS peak detection, including using minimum height, signal-to-noise, width, and area [13]. Compounds were identified by cross-comparison to library entries of purified standards that contains the retention time/index (RI), mass to charge ratio (*m/z*), and chromatographic data (including MS/MS spectral data). The MS/MS scores are based on a comparison of the ions present in the experimental spectrum to the ions present in the library spectrum. There are more than 3300 commercially purified standard compounds registered in Metabolon Laboratory Information Management System for distribution to LC-MS platform for determination of their analytical characteristics. Each rice bran metabolite was then cross-checked for an associated number in the Kyoto Encyclopedia of Genes and Genomes (KEGG), Human Metabolome Database (HMDB), and PubChem databases. 

### 4.5. Metabolic Pathway Analysis

Across all 17 rice bran varieties, the metabolome analysis comprised 53 metabolic pathways, and each metabolite was assigned to one pathway. Using the following equation, pathway enrichment score (PES) was calculated, where “*k*” correspond to the number of metabolites with Z-score of ±2 or larger in a metabolic pathway, “*m*”, corresponding to the total number of metabolites identified in that pathway, “*n*”, correspond to the total number of significant metabolites in the dataset, and “*N*” corresponds to the total number of identified metabolites in the entire dataset:

$$\frac{k/m}{n/N}$$

Metabolic pathways that had a PES of less then or greater than 1 indicated that the pathway contained one or more differentially expressed metabolites, compared to all other pathways.

### 4.6. Rice Biosynthetic Gene Identification for Selected Rice Bran Metabolites

The *Oryza sativa* (japonica group-based) OryzaCyc 4.0, Plant Metabolic Network (PMN) database was used to identify rice biosynthetic genes that produce selected bran metabolites that were involved in human and animal health promotion. Bran metabolites selected for rice gene analysis had Z-score of ±2 or larger when comparing the 17 cultivars in the metabolomics dataset. The rice gene and bran metabolite linkages can be verified at (http://plantcyc.org/databases/oryzacyc/4.0). It is notable that a limitation arises from the fact that the rice genome database is limited to japonica sub-population, and it may differ for the indica sub-population [96]. 

### 4.7. Statistical Analysis

Median-scaled relative abundance and Z-score for each of the metabolites and across all cultivars were calculated as previously described [13]. Z-score calculation was based on median-scaled relative abundances and reported as standard deviations from the mean, and it was calculated using the following formula:

$$Z\;=\;\frac{x-\mu }{\sigma }$$

where relative abundance of the metabolite is expressed as “*x*”, the mean of relative abundance for the metabolite across 17 rice brans is expressed as “μ”, and the relative abundance standard deviation of same metabolite across 17 cultivars is expressed as “σ”. A notable Z-score for a metabolite shows that the relative abundance of that metabolite in a specific cultivar is lower or higher than the standard deviations from the mean of other cultivars. For each variety, metabolites with a Z-score that is greater than 2 or that is less than −2 (Z-score > **|**2.0**|**) were considered to be distinguishers of that variety. Furthermore, a principal component analysis (PCA) was completed using SIMCA (Sartorius Stedim Biotech) to assess the overall variability in the global metabolite profile of bran from 17 rice cultivars using median-scaled relative abundance values. However, the country in which the rice cultivar originated from or was grown in was not a variable in the PCA as the means to focus differences in rice varieties that are available to consumers in various countries.

## Figures and Tables

**Table 1:** Number of rice bran metabolites with confirmed annotation in each cultivar by cultivar and chemical class.

	Rice Cultivar	Kenya—Basmati 217	Kenya—Basmati 370	Mali—Gambiaka *	Mali—Shwetasoke	Mali—DM-16	Mali—Khao Gaew	Nicaragua—Dorado	Nepal—Sawa Mahsuli	India—Chennula	India—Njavara *	USA—Calrose	USA—RBT 300	USA—Jasmine 85	USA—IAC 600	USA—LTH	USA—SHZ-2	Cambodia —Rang Jey
Chemical Class																		
Amino acids		119	117	120	117	115	99	117	117	105	99	119	122	110	113	119	117	108
Carbohydrates		51	52	53	53	52	50	51	50	51	50	48	49	50	53	53	50	51
Cofactors & vitamins		27	27	27	27	26	24	27	27	27	26	27	23	27	27	27	26	27
Lipids		166	165	165	166	159	164	161	155	155	151	165	161	146	163	165	150	156
Nucleotides		37	38	35	35	34	29	37	38	30	29	35	33	37	36	35	36	36
Peptides		0	2	11	11	11	7	3	11	1	1	11	1	11	10	8	11	2
Secondary metabolites		19	21	19	19	20	19	16	20	19	22	16	17	17	20	22	17	21
Total		419	422	430	428	417	392	412	418	388	378	421	406	398	422	429	407	401

* notes the cultivars with the largest and smallest numbers of identified metabolites.

**Table 2:** Metabolite–rice gene relationships identified from OryzaCyc (*Oryza sativa* japonica group), Plant Metabolic Network (PMN) database.

Rice Bran Metabolites	Precursor	Biosynthesis Pathway	No. of Genes *	Gene ID	Gene(s) Name
**Amino Acids**					
**Aromatic amino acids (phosphoenolpyruvate (PEP)-derived)**					
l-quinate	Trans-5-*O*-caffeoyl-d-quinate	Caffeoylglucarate biosynthesis	5	GN7F-30156	LOC_Os02g39170.1
				GN7F-16973	LOC_Os02g39590.1
				GN7F-15850	LOC_Os06g47910.1
Serotonin	Tryptamine	Hydroxycinnamic acid Serotonin amides biosynthesis	2	GN7F-19639	LOC_Os08g04560.1
				GN7F-25663	LOC_Os08g04540.1
Tryptophan	l-serine	Tryptophan biosynthesis	4+ (153)	GN7F-27027	LOC_Os03g58260.1
				GN7F-24368	LOC_Os03g58290.1
				GN7F-25293	LOC_Os06g42560.4
				GN7F-19428	LOC_Os08g04180.1
Tyrosine	l-phenylalanine, l-arogenate	Phenylalanine degradation V, tyrosine biosynthesis II & III	3+ (153)	GN7F-27976	LOC_Os06g35050.1
				GN7F-19057	LOC_Os06g49505.1
				GN7F-18001	LOC_Os06g49520.1
**Aspartate family (oxaloacetate (OAA)-derived)**					
Asparagine	l-aspartate, 3-cyano-l-alanine	Asparagine biosynthesis I & II, cyanide detoxification I	5+ (153)	GN7F-32447	LOC_Os12g38630.1
				GN7F-23509	LOC_Os06g15420.1
				GN7F-23610	LOC_Os03g18130.1
				GN7F-15965	LOC_Os02g42350.1
				GN7F-23159	LOC_Os02g42330.1
Aspartate	l-asparagine, 3-cyano-l-alanine, indole-3-acetyl-aspartate-*N*-β-d-glucose	Asparagine degradation I, cyanide detoxification I, Indole-3-acetate conjugate biosynthesis II	3+ (153)	GN7F-15965	LOC_Os02g42350.1
				GN7F-23159	LOC_Os02g42330.1
				GN7F-27949	LOC_Os04g58600.2
Lysine	Meso-diaminopimelate	Lysine biosynthesis VI	1+ (153)	GN7F-25633	LOC_Os02g24354.1
Methionine sulfoxide	An acyl-CoA, an aldehyde, a carboxylic ester	Not in pathway	9+ (150)	GN7F-28729	LOC_Os09g34190.1
				GN7F-19329	LOC_Os04g47120.1
				GN7F-31811	LOC_Os01g12910.1
				GN7F-31843	LOC_Os07g27870.1
				GN7F-32115	LOC_Os04g35590.1
				GN7F-32205	LOC_Os07g27960.1
				GN7F-32376	LOC_Os02g32200.1
				GN7F-32723	LOC_Os01g12920.1
				GN7F-32765	LOC_Os01g65950.1
Threonine	*O*-phospho-l-homoserine, l-threonine 3-*O*-phosphate	Threonine biosynthesis from homoserine, l-threonine 3-*O*-phosphate	3+ (153)	GN7F-18835	LOC_Os01g49890.1
				GN7F-30196	LOC_Os05g47640.1
				GN7F-29436	LOC_Os08g17784.1
**Glutamate family (α-ketoglutarate-derived)**					
Arginine	l-arginino-succinate	Arginine biosynthesis I & II, Citrulline-nitric oxide cycle	3+ (153)	GN7F-20973	LOC_Os03g19280.1
				GN7F-32460	LOC_Os03g60976.1
				GN7F-32707	LOC_Os03g60992.1
Glutamate ^1^	More than 35 precursors (top two: l-glutamine, 2-oxoglutarate)	More than 27 pathways (top two: 4-aminobenzoate biosynthesis, 4-aminobutyrate degradation)	91+ (157)	GN7F-17849	LOC_Os06g48620.1
				GN7F-25902	LOC_Os04g52440.1
				GN7F-28339	LOC_Os08g10510.1
				GN7F-27233	LOC_Os02g02210.1
				GN7F-19896	LOC_Os04g52450.1
Glutamine	More than eight precursors (top three: l-glutamate, a dipeptide with proline at the C-terminal, a γ l-glutamyl-l-amino acid)	More than six pathways (top four: Ammonia assimilation cycle I & II, glutamine biosynthesis I & II)	5+ (153)	GN7F-15709	LOC_Os03g50490.1
				GN7F-15901	LOC_Os04g56400.1
				GN7F-22516	LOC_Os03g12290.1
				GN7F-26393	LOC_Os10g31820.1
				GN7F-27460	LOC_Os02g50240.1
*N*-acetylglutamate	l-glutamate	Arginine biosynthesis II (acetyl cycle), ornithine biosynthesis	6+ (150)	GN7F-20894	LOC_Os03g17120.1
				GN7F-19328	LOC_Os07g39690.1
				GN7F-17187	LOC_Os03g31690.1
				GN7F-31311	LOC_Os03g46200.1
				GN7F-32148	LOC_Os03g58010.1
				GN7F-32821	LOC_Os03g58030.1
Pyroglutamine	An (γ-l-glutamyl)-l-amino acid	γ-glutamylcyclotransferase	2	GN7F-31386	LOC_Os03g63700.1
				GN7F-32110	LOC_Os11g04420.4
**Serine family (phosphoglycerate-derived)**					
Glycine	l-cysteinyl-glycine	γ-glutamyl cycle, Phytochelatins biosynthesis	8+ (151)	GN7F-30607	LOC_Os01g05810.1
				GN7F-16957	LOC_Os04g38450.1
				GN7F-25454	LOC_Os01g05820.1
				GN7F-26619	LOC_Os05g34290.1
				GN7F-28075	LOC_Os06g01260.1
				GN7F-16002	LOC_Os12g35890.1
				GN7F-26733	LOC_Os09g32290.2
				GN7F-19516	LOC_Os01g21380.1
Taurine ^2^	In transport reactions		4	GN7F-31312	LOC_Os09g29660.1
				GN7F-31505	LOC_Os05g31080.1
				GN7F-32616	LOC_Os03g20170.1
				GN7F-32729	LOC_Os09g29670.1
**Lipids**					
**Free fatty acid**					
α-linolenate	A phosphatidylcholine	No common pathways	1+ (334)	GN7F-18386	LOC_Os11g04940.1
Laurate	Lauroyl-CoA, a dodecanoyl-[acyl-carrier protein]	Palmitate biosynthesis II (bacteria and plants), sporopollenin precursors biosynthesis	1+ (285)	GN7F-19329	LOC_Os04g47120.1
Linoleate	Linoleoyl-CoA	No common pathways	2+ (332)	GN7F-33079	LOC_Os02g44654.2
				GN7F-16232	LOC_Os04g47250.1
Myristate	Myristoyl-CoA	No common pathways	0+ (325)	-	-
Palmitate	Palmitoyl-CoA, a palmitoyl-[acp], 1,2-dipalmitoyl-phosphatidylcholine, 1-palmitoyl-2-linoleoyl-phosphatidylcholine	Cutin biosynthesis, sporopollenin precursors biosynthesis, suberin monomers biosynthesis, palmitate biosynthesis II (bacteria and plants), phospholipid remodeling (phosphatidylcholine, yeast)	6+ (333)	GN7F-19329	LOC_Os04g47120.1
				GN7F-28729	LOC_Os09g34190.1
				GN7F-32532	LOC_Os01g66240.1
				GN7F-24830	LOC_Os01g46250.1
				GN7F-20654	LOC_Os01g73740.1
				GN7F-18158	LOC_Os01g51360.1
Palmitoleate	A palmitoleoyl-[acyl-carrier protein]	No common pathways	4+ (333)	GN7F-31362	LOC_Os04g46710.1
				GN7F-31587	LOC_Os04g46730.1
				GN7F-31765	LOC_Os02g44134.1
				GN7F-32681	LOC_Os02g44200.1
**Oxylipins**					
9,10-epoxystearate	Oleate, a hydroperoxy-fatty-acyl-[lipid]	Cutin biosynthesis, poly-hydroxy fatty acid biosynthesis	10+ (332)	GN7F-31064	LOC_Os10g37070.1
				GN7F-30659	LOC_Os08g05620.1
				GN7F-18468	LOC_Os01g24810.1
				GN7F-27809	LOC_Os10g05020.1
				GN7F-16745	LOC_Os04g03890.1
				GN7F-21459	LOC_Os10g37100.1
				GN7F-19209	LOC_Os04g33370.1
				GN7F-18601	LOC_Os02g01890.1
				GN7F-29035	LOC_Os08g05610.1
				GN7F-15971	LOC_Os06g46680.1
**Phospholipid Metabolism**					
Glycerophosphorylcholin (sn-glycero-3-phosphocholine)	A 1-acyl-sn-glycero-3-phosphocholine	No common pathways	7	GN7F-33055	LOC_Os01g07960.3
				GN7F-29598	LOC_Os04g57370.1
				GN7F-18763	LOC_Os04g09540.1
				GN7F-31398	LOC_Os01g42690.1
				GN7F-32181	LOC_Os04g57390.1
				GN7F-32498	LOC_Os05g51050.1
				GN7F-32638	LOC_Os08g42680.1

* Numbers in brackets indicate the number of common genes known to be responsible for the synthesis of a standard alpha amino acid and a standard fatty acid. ^1^ Only the top five genes (most commonly studied) were included in the table. ^2^ There are no identified genes for taurine biosynthesis in rice. However, genes that are responsible for its transport from environment into the rice plant are identified.

**Table 3:** Metabolite–rice gene relationships identified from OryzaCyc (*Oryza sativa* japonica group), Plant Metabolic Network (PMN) database.

Rice Bran Metabolites	Precursor	Biosynthesis Pathway	No. of Genes *	Gene ID	Gene(s) Name
**Carbohydrates**					
**Citric acid cycle (TCA) cycle**					
α-ketoglutarate (2-oxoglutarate) ^1^	l-glutamate, d-*threo*-isocitrate	Nine pathways (top four: Alanine degradation II, Glutamate degradation I, Arginine biosynthesis II, Ornithine biosynthesis)	11+ (160)	GN7F-23833	LOC_Os03g58040.1
				GN7F-16177	LOC_Os02g43470.1
				GN7F-28651	LOC_Os04g45970.1
				GN7F-23252	LOC_Os05g03830.1
				GN7F-26515	LOC_Os07g27780.1
*cis*-aconitate	Citrate	Glutamine biosynthesis III glyoxylate cycle, TCA cycle II (plants and fungi)	4+ (150)	GN7F-24702	
				GN7F-28499	LOC_Os10g03960.1
				GN7F-23691	LOC_Os06g19960.1
				GN7F-25422	LOC_Os03g04410.1
				GN7F-25644	LOC_Os08g09200.1
Malate	Acetyl-CoA, Glyoxylate, Fumarate	Glycolate and glyoxylate degradation II, glyoxylate cycle, superpathway of glyoxylate cycle and fatty acid degradation, TCA cycle II (plants and fungi)	2+ (150)	GN7F-24000	LOC_Os04g40990.1
				GN7F-21211	LOC_Os03g21950.1
**Cofactors & vitamins**					
**Tocopherol metabolism**					
β-tocopherol	δ-tocopherol, *S*-adenosyl-l-methionine	Vitamin E biosynthesis (tocopherols)	4	GN7F-31982	LOC_Os10g41970.1
				GN7F-31239	LOC_Os03g26200.1
				GN7F-31334	LOC_Os08g02600.1
				GN7F-25500	LOC_Os02g47310.1
γ-tocotrienol	2,3-dimethyl-6-geranylgeranyl-1,4-benzoquinol	Vitamin E biosynthesis (tocopherols)	1	GN7F-24601	LOC_Os02g17650.1

* Numbers in brackets indicate the number of common genes known to be responsible for the synthesis of a standard carbohydrate. ^1^ Only the top five genes were included in the table.

**Table 4:** Metabolite-rice gene relationship identified from OryzaCyc (*Oryza sativa* japonica group), Plant Metabolic Network (PMN) database.

Rice Bran Metabolites	Precursor	Biosynthesis Pathway	No. of Genes *	Gene ID	Gene(s) Name
**Nucleotides**					
**Purine metabolism**					
Adenine ^1^	*S*-methyl-5′-thioadenosine, adenosine, *N6*-dimethylallyladenine, *trans*-zeatin, *cis*-zeatin, *N1*-ethyladenine, *N1*-methyladenine	*S*-methyl-5′-thioadenosine degradation I, adenine and adenosine salvage II, cytokinins degradation	18	GN7F-25353	LOC_Os08g44370.1
				GN7F-26929	LOC_Os09g39440.1
				GN7F-32781	LOC_Os05g33644.1
				GN7F-32797	LOC_Os05g33630.1
				GN7F-19530	LOC_Os06g37500.1
Adenosine	*S*-adenosyl-l-homocysteine, trans-zeatin riboside, isopentenyl adenosine	*S*-adenosyl-l-methionine cycle II, l-methionine degradation I (to l-homocysteine), cytokinins degradation	3+ (8)	GN7F-20280	LOC_Os02g12780.1
				GN7F-19530	LOC_Os06g37500.1
				GN7F-20388	LOC_Os01g09260.1
Adenosine 5′-monophosphate ^1^	Adenosine triphosphate (ATP)	More than 97 pathways (top four: Trans-zeatin biosynthesis, Adenosine nucleotides degradation I, 4-hydroxybenzoate biosynthesis I (eukaryotes), l-arginine biosynthesis I (via l-ornithine)	299+ (24)	GN7F-23647	LOC_Os02g46970.1
				GN7F-23504	LOC_Os06g44620.1
				GN7F-28551	LOC_Os08g34790.1
				GN7F-25622	LOC_Os08g14760.1
				GN7F-25996	LOC_Os01g24030.1
Hypoxanthine	Inosine	Adenosine nucleotide degradation I	5	GN7F-25353	LOC_Os08g44370.1
				GN7F-21573	LOC_Os03g31170.1
				GN7F-26929	LOC_Os09g39440.1
				GN7F-32781	LOC_Os05g33644.1
				GN7F-32797	LOC_Os05g33630.1
**Secondary metabolites**					
**Benzenoids**					
Salicylate	Methylsalicylate	Unknown	3 + (152)	GN7F-26541	LOC_Os05g30760.1
				GN7F-21475	LOC_Os01g37650.1
				GN7F-28107	LOC_Os01g25360.1

* Numbers in brackets indicate the number of common genes known to be responsible for biosynthesis of a nucleotide or a carboxylate. ^1^ Only the top five genes were included in the table.

**Table 5:** Classification and phenotypes of the 17 rice cultivars used for bran metabolomics.

Cultivar	Grain Type	Bran Color	Country of Origin	Growing Location
**Basmati 217**	Long	Brown	India	Kenya
**Basmati 370**	Long	Brown	India	Kenya
**Calrose**	Medium	Brown	USA	California
**Chennula**	Long	Brown	India	India
**DM-16**	Short	Brown	South America	Mali
**Dorado**	Long	Brown	Colombia	Nicaragua
**Gambiaka**	Long	Brown	Mali	Mali
**IAC 600**	Medium	Purple	Brazil	Arkansas
**Jasmine 85**	Long	Brown	Philippines	Arkansas
**Khao Gaew**	Long	Brown	Thailand	Mali
**Li-Jiang-Xin-Tuan-Hei-Gu (LTH)**	Medium	Red	China	Arkansas
**Njavara**	Long	Red	India	India
**Rang Jey**	Medium	Brown	Cambodia	Cambodia
RBT 300 *	Medium	Brown	USA	California
**Sawa Mahsuli**	Long	Brown	Nepal	Nepal
**Shan-Huang-Zhan-2 (SHZ-2)**	Long	Brown	China	Arkansas
**Shwetasoke**	Long	Brown	Mali	Mali

* This rice bran is a commercial ingredient from a mixture of varieties grown in California.

## Supplementary Materials

The following are available online at http://www.mdpi.com/2218-1989/8/4/63/s1, Supplementary Table S1: List of all the identified metabolites in bran of 17 rice cultivars with their relative abundances, Supplementary Table S2: Z-scores and relative abundances of 71 significant metabolites, Supplementary Table S3: Metabolic pathway enrichment scores (PES) for discriminating metabolites between rice bran varieties.
