# Supplementary material for: Comparative Rice Bran Metabolomics across Diverse Cultivars and Functional Rice Gene–Bran Metabolite Relationships
Source: Metabolites. 2018 Oct 9;8(4):63. doi: 10.3390/metabo8040063 (PMC6315861; doi:10.3390/metabo8040063)
Supplement: Supplementary file 1 [file metabolites-08-00063-s001.zip › Supplementary Table 3_PES_Significant sub-pathways.pdf]

Supplementary Table 3. Metabolic pathway enrichment scores (PES) for discriminating metabolites between rice bran varieties

Chemical class	Metabolic Pathway	Kenya – Basmati 217	Kenya – Basmati 370	Mali – Gambiaka	Mali – Shwetasoke	Mali – DM-16	Mali – Khao Gaew	Nicaragua – Dorado	Nepal – Sawa Mahsuli	India – Chennula	India – Njavara	USA – Calrose	USA – RBT 300	USA – Jasmine 85	USA – IAC 600	USA – LTH	USA – SHZ-2	Cambodia – Rang Jey
Amino acid	**Aromatic amino acid metabolism (PEP derived)**	1.0	1.0	1.0	1.0	**24.8**	1.0	1.0	1.0	1.0	**1.7**	1.0	1.0	1.0	1.0	1.0	1.0	**2.4**
	**Aspartate family (OAA derived)**	1.0	1.0	1.0	1.0	1.0	**1.6**	1.0	**1.2**	**3.9**	**1.1**	1.0	1.0	1.0	**1.5**	1.0	1.0	1.0
	**Glutamate family (alpha-ketoglutarate derived)**	1.0	1.0	**6.9**	1.0	1.0	1.0	1.0	1.0	**0.9**	**2.1**	1.0	1.0	1.0	1.0	1.0	1.0	1.0
	**Serine family (phosphoglycerate derived)**	1.0	1.0	1.0	1.0	1.0	1.0	1.0	**6.0**	1.0	**2.9**	1.0	1.0	1.0	1.0	1.0	1.0	1.0
Carbohydrate	**Amino sugar and nucleotide sugar**	**2.9**	1.0	1.0	1.0	1.0	**20.3**	1.0	1.0	1.0	**0.7**	1.0	1.0	1.0	1.0	1.0	1.0	1.0
	**TCA cycle**	1.0	1.0	1.0	1.0	1.0	1.0	1.0	1.0	1.0	**9.9**	**7.4**	1.0	1.0	**6.8**	1.0	1.0	**1.2**
Cofactors & vitamins	**Tocopherol metabolism**	1.0	1.0	1.0	1.0	1.0	1.0	1.0	**9.6**	1.0	1.0	1.0	1.0	1.0	1.0	1.0	1.0	1.0
Lipids	**Free fatty acid**	1.0	1.0	1.0	1.0	1.0	1.0	1.0	1.0	**2.5**	1.0	1.0	**3.1**	1.0	1.0	1.0	1.0	1.0
	**Glycerolipids (diacyl)**	1.0	1.0	1.0	1.0	1.0	1.0	1.0	1.0	**1.2**	1.0	1.0	**12.8**	**4.1**	1.0	1.0	1.0	**2.1**
	**Glycerolipids (monoacyl)**	1.0	1.0	1.0	1.0	1.0	1.0	1.0	1.0	**3.6**	1.0	1.0	**5.7**	1.0	1.0	1.0	1.0	1.0
	**Lyso-phospholipids**	1.0	1.0	1.0	1.0	1.0	1.0	1.0	1.0	1.0	1.0	1.0	1.0	1.0	1.0	1.0	1.0	**3.6**
	**Oxylipins**	1.0	1.0	1.0	1.0	1.0	1.0	1.0	1.0	1.0	1.0	1.0	**6.5**	1.0	1.0	1.0	1.0	1.0
	**Phospholipid Metabolism**	1.0	1.0	1.0	1.0	1.0	1.0	1.0	1.0	1.0	**4.6**	1.0	1.0	1.0	1.0	1.0	1.0	1.0
Nucleotide	**Purine metabolism**	1.0	1.0	1.0	1.0	1.0	**1.1**	1.0	1.0	1.0	1.0	1.0	**1.4**	1.0	1.0	1.0	1.0	**2.7**
Secondary metabolism	**Benzenoids**	1.0	1.0	1.0	1.0	1.0	**3.2**	1.0	1.0	1.0	1.0	1.0	1.0	1.0	1.0	1.0	1.0	**4.3**

PES greater or less than 1.0 is notable (bolded) and it indicates that the metabolic pathway contains at least 1 metabolite with significant Z-score (Z-score > |2.0|), i.e., level of expression for that metabolite is different from the rest of cultivars for the same metabolites across the metabolic pathway.
